# Case Report: Surgical management of giant multiple aneurysms after Kawasaki disease in a teenager

**DOI:** 10.3389/fped.2025.1622729

**Published:** 2025-09-04

**Authors:** Giuseppe Fischetti, Lorenzo Giovannico, Giovanni Meliota, Nicola Di Bari, Domenico Parigino, Elena Massari, Maristella Lombardi, Ugo Vairo, Tomaso Bottio, Massimo A. Padalino

**Affiliations:** ^1^ Cardiac Surgery Unit, Department of Precision and Regenerative Medicine and Jonian Area, University of Bari, Bari, Italy; ^2^ Pediatric Cardiology Unit, Department of Pediatrics, Ospedale Pediatrico Giovanni XXIII, Bari, Italy; ^3^ Congenital Heart Surgery Unit, Department of Precision and Regenerative Medicine and Jonian Area, University of Bari, Bari, Italy

**Keywords:** Kawasaki disease, coronary, surgery, outcome, bypass

## Abstract

Kawasaki disease (KD), or mucocutaneous lymph node syndrome, is a rare systemic inflammatory condition predominantly affecting children under 5 years of age. Complications such as giant coronary artery aneurysms, although rare due to advancements in treatment, remain life-threatening. Coronary artery bypass grafting (CABG) has been a well-established treatment for severe coronary lesions caused by KD. In rare cases of ischemic cardiomyopathy in pediatric patients, heart transplantation may be the only option. We report a case of a 15-year-old male with a history of KD diagnosed at 9 months of age, complicated by giant coronary aneurysms of the left anterior descending and right coronary arteries, who underwent a successful double CABG using the left internal mammary artery) and a saphenous vein graft.

## Background

Kawasaki disease (KD), also known as mucocutaneous lymphnode syndrome, is a rare acute systemic inflammatory condition that predominantly affects children under the age of five (1). It primarily targets medium-sized blood vessels and has an incidence of approximately 0.25 per 1,000 children (2). The development of aneurysms represents the most severe complication of the disease. Advances in medical treatment over the years have significantly reduced the occurrence of giant coronary artery aneurysms, lowering the rate to 0.2%–0.3%.

More than 40 years have passed since Kitamura et al. (3) first reported a coronary artery bypass grafting (CABG) procedure performed on a child with KD. Since then, CABG has become the standard surgical intervention for managing coronary lesions in children with KD due to its excellent clinical outcomes. However, in cases of severe ischemic cardiomyopathy, heart transplantation remains the only viable treatment option.

Here, we present the case of a teenager with multiple gigantic aneurysms, successfully treated with surgical coronary artery bypass grafting.

## Case report

**Figure 1:**
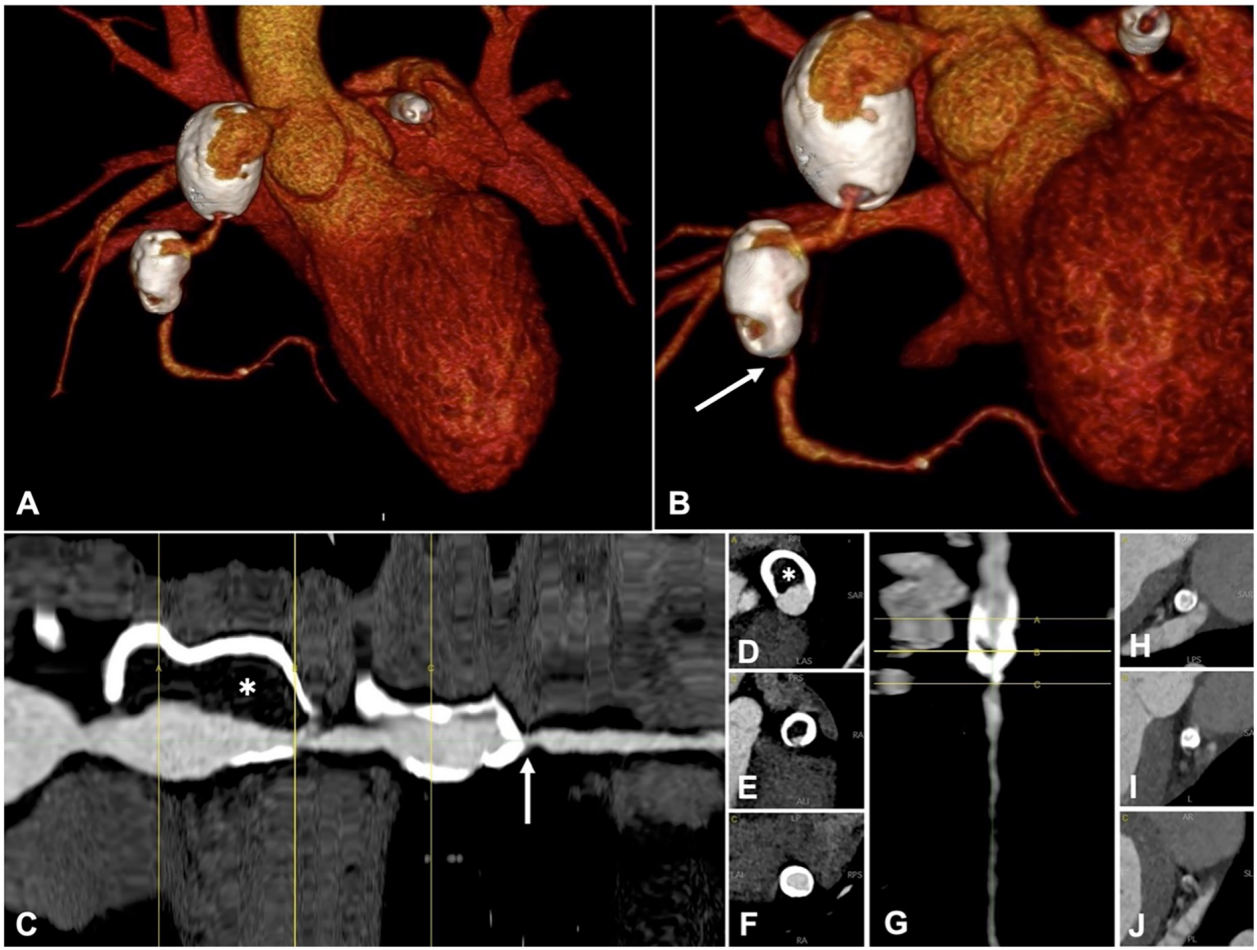
Ct coronary angiography, one year before index acute coronary syndrome. A-B: 3D volume rendering, anterior-posterior view **(A)** and caudal left anterior oblique view **(B)** Right coronary artery (RCA) has two giant aneurysms, with extensive parietal calcifications. **(C–G)** straightened curved-mutiplanar reconstruction (cMPR) of the RCA **(C)** and LAD **(G)**, with sequential cross sections of RCA **(D–F)** and LAD **(H–J)**. Lumen diameter is irregular, with dilations alternating to stenoses. Critical stenosis of the mid RCA is shown (arrow). There is a high thrombotic burden within the proximal RCA aneurysm (asterisk). cMPR shows a critical stenosis at the caudal portion of LAD aneurysm **(G–J)**.

We report the case of a 15-year-old male patient with an unremarkable perinatal history who was diagnosed with Kawasaki disease (KD) at 9 months of age. At the time of diagnosis in infancy, he was treated with a single dose of intravenous immunoglobulin (2 g/kg administered over 12 h) and high-dose acetylsalicylic acid (80 mg/kg/day in divided doses), in accordance with standard first-line therapy recommendations. This regimen is intended to reduce systemic inflammation and prevent coronary artery complications. Despite appropriate initial treatment, he subsequently developed multiple giant coronary artery aneurysms involving the left anterior descending (LAD) and right coronary arteries (RCA), necessitating long-term oral anticoagulation therapy.During follow-up at multiple tertiary centers, the aneurysms did not regress in size. Periodic coronary angiography (CA) and computed tomography (CT) revealed parietal calcifications and thrombotic stratifications (Figure 1). Stress cardiac magnetic resonance imaging (stress CMR) demonstrated inducible ischemia in the apical segment of the anterolateral left ventricular (LV) wall, accounting for less than 10% of the LV mass. Consequently, the patient was placed on triple antithrombotic therapy consisting of aspirin, clopidogrel, and warfarin. Frequent episodes of isolated ventricular ectopy were successfully managed with metoprolol.

At age 14, the patient experienced a prolonged episode of chest pain accompanied by a mild elevation of troponin levels. At the time, the international normalized ratio (INR) was below the therapeutic range. CT imaging showed an increased thrombotic burden within the giant RCA aneurysm, as well as severe stenosis of the mid-RCA and proximal LAD. Triple antithrombotic therapy was optimized, and statins were introduced to the regimen.

**Figure 2:**
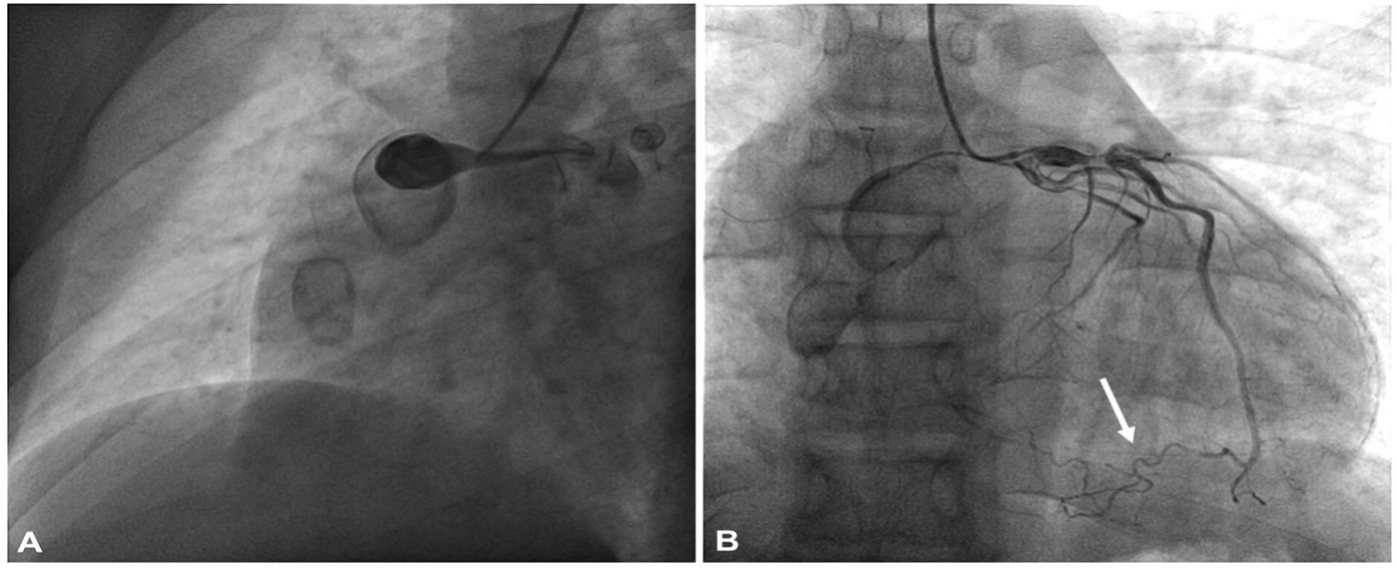
Selective coronary angiography **(A)** right coronary artery angiography showing total occlusion within the proximal aneurism; **(B)** angiography of left coronary artery showing aneurism in the proximal portion and a severe stenosis distal to the first septal branch origin. Collateral circulation from the LAD to the distal RCA-posterior descending artery (inferior arrow).

One year later, at age 15, the patient presented to our emergency department with new-onset chest pain. On arrival, the electrocardiogram (ECG) revealed slight ST-segment depression in leads V4, V5, and V6, as well as T-wave inversions. High-sensitivity troponin I levels were elevated to 300 ng/ml. The INR remained below the therapeutic range (1.7). A 2D echocardiogram demonstrated hypokinesia of the inferior and posterolateral LV walls, while global left ventricle ejection fraction (LVEF) was preserved at 60%. A CT scan performed one year earlier was utilized to reconstruct the patient's coronary anatomy in 3D before performing CA (Figures 1A,B). Selective angiographies revealed occlusion of RCA within the proximal aneurysm, with collateral circulation from the LAD to the distal RCA-posterior descending artery. A severe stenosis of proximal LAD was confirmed (Figure 2).

**Figure 3:**
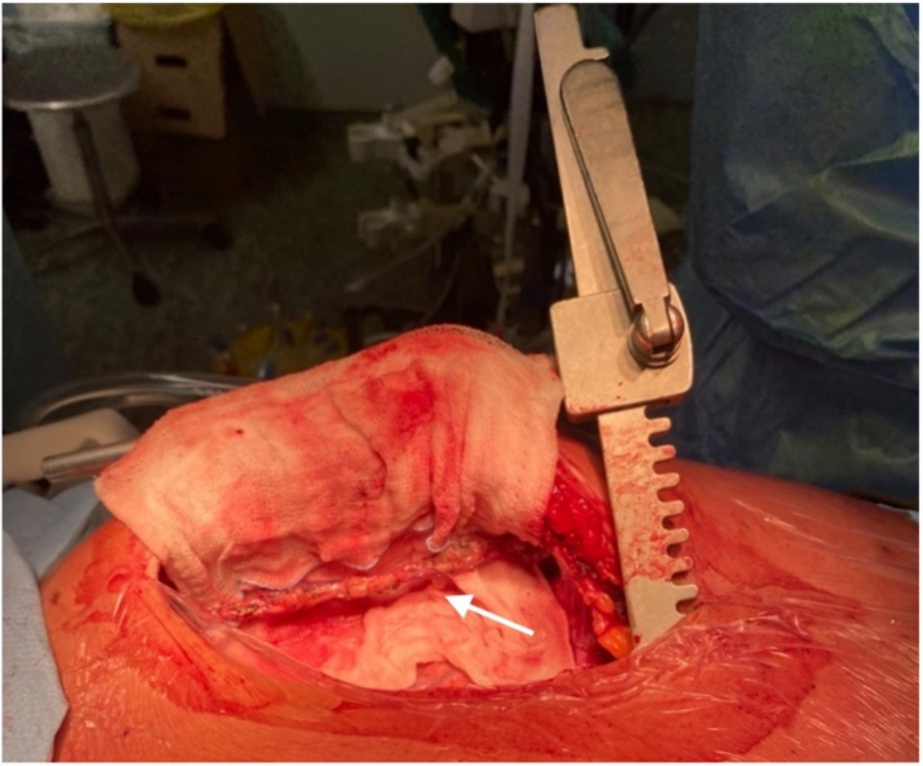
LIMA harvesting (see arrow).

**Figure 4:**
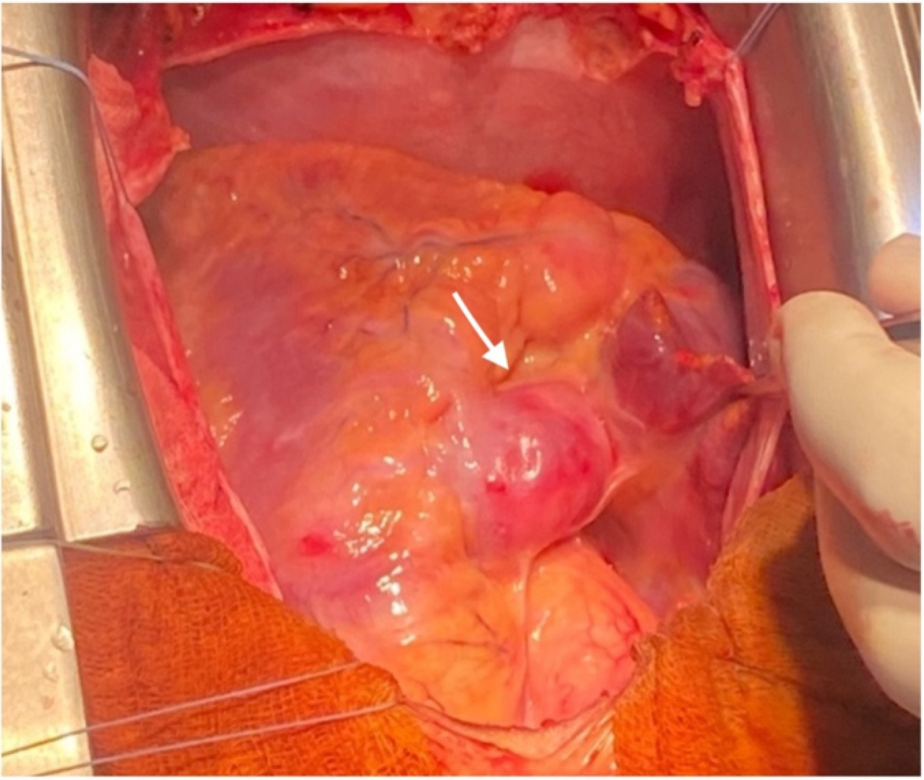
Intraoperative image: giant aneurysm of right coronary artery (see arrow). The right atrial appendage is lifted to expose right coronary aneurysm.

Following a multidisciplinary Heart Team discussion, it was concluded that the patient would benefit from double coronary artery bypass grafting. After comprehensive counseling of the family, including a detailed explanation of the potential benefits and risks of the procedure, the patient was admitted for elective surgery. A median longitudinal sternotomy (Figures 3, 4) was performed, followed by the harvesting of the left internal mammary artery (LIMA) (Figures 3 and 4) and a segment of the saphenous vein (SVG). The patient's high thrombotic burden and unstable INR levels influenced surgical planning. Full heparinization and careful graft handling were used to minimize embolic risk. On cardiopulmonary bypass and with aortic cross-clamping, the distal anastomosis of the SVG to the posterior interventricular artery and the end-to-side anastomosis to the left anterior descending artery (LAD) were completed. Additionally, under tangential clamping on a beating heart, the proximal anastomosis of the venous graft was performed. The patient was successfully weaned off cardiopulmonary bypass uneventfully.

Upon transfer to the postoperative care unit, the patient was extubated early and closely monitored for 24 h. His postoperative recovery was uneventful, and he was discharged on postoperative day 6 with a regimen of acetylsalicylic acid, apixaban, atorvastatin, and metoprolol. At 12-months follow-up, the patient is in good clinical conditions, NYHA class 1, with preserved global LVEF (65%), and no evidence of LV wall motion abnormalities, and the 24-h Holter ECG is unremarkable. A follow-up coronary computed tomography angiography is scheduled at 24 months.

## Discussion

This report describes a rare case of a 15-year-old boy presenting with aneurysmal complications of Kawasaki disease (KD), initially diagnosed at nine months of age, successfully treated with double CABG. Diagnostic imaging included echocardiography, cardiac CT scans, and coronary angiography.

Revascularization was urged because of a recurrent episode of acute coronary syndrome, characterized by total thrombotic occlusion of the RCA and critical stenosis of the proximal LAD coronary artery. Polyurethane-covered stents have recently been proposed as an emerging option for managing coronary aneurysms, offering potential exclusion of aneurysmal segments while preserving distal flow. However, their use in pediatric patients remains limited, and long-term safety and efficacy are yet to be established (4). Furthermore, the 2017 American Heart Association (AHA) guidelines recommend CABG over percutaneous coronary intervention (PCI) in older children and adults with KD and multivessel involvement (6). Thus, we opted for a more traditional CABG revascularization in our young patient. The timing of surgery was influenced by several factors, including the degree of coronary artery stenosis, condition of collateral circulation, severity of myocardial ischemia, surgical expertise, and patient/family understanding of the procedure (7).

Current literature highlights various conduits for CABG, including the gastroepiploic artery, LIMA, SVGs, and radial artery. Although CABG is generally safe in KD, the arterial revascularization with LIMA shows excellent long-term patency and growth potential (5). Consequently, it is usually the preferred graft choice for pediatric patients with Kawasaki disease-related coronary artery complications.

In our patient, a single LIMA bypass to the LAD was initially planned. However, during surgery, the feasibility of an additional bypass to the posterior interventricular branch (PIV) was recognized. Since the right internal mammary artery (RIMA) was too short to bypass the RCA aneurysms, and to minimize cardiopulmonary bypass (CPB) time, a saphenous vein graft (SVG) was harvested for this purpose (8), with an excellent early outcome.

Despite not optimal, use of SVGs for CABG in Kawasaki disease has been reported since 1975, but long-term outcomes beyond 20 years are not well-documented. A study assessing the patency of SVGs in 13 adult patients (20 grafts) reported patency rates of 84.4%, 57.2%, and 51.5% at 1, 10, and 25 years, respectively. Minor irregularities in SVG walls were noted in some long-term cases. Selected patients have demonstrated long-term patency exceeding 20 years, underscoring the importance of aggressively managing risk factors such as obesity, hypertension and hyperlipidemia (9). However, it is well known that right and left coronary systems are connected via collateral vessels, that can connect either a proximal to a distal section of the same coronary artery or one coronary artery to another. When a coronary (or graft) stenosis occurrs, the resultant decrease in distal coronary pressure produces a pressure gradient across the collateral network leading to a marked increase in collateral blood flow and vessel diameter (10). Thus, as experienced in children and young patients after arterial switch operation for transposition of the great arteries (11), the chronic slow coronary (or SVGs) graft occlusion may be silent thanks to the gradual development of coronary collateral vessels that can occur especially in young patients. As such, we reccommend a reinforced clinical surveillance, with periodical function testing, tailored to the patient's individual anatomic characteristics.

Last but not least, in our patient both the previous acute coronary episodes were likely to be triggered by subtherapeutic INR values, caused by patient reduced compliance, and resulting in acute thrombosis within giant aneurysms and subsequent myocardial ischemia. In consideration of this, and the presence of SVG post CABG, we chose a postoperative chronic anticoagulation regimen with apixaban, which has proven to be effective in thrombosis prevention in children with cardiac disease, with an acceptable safety profile regarding bleeding risk (12).

## Conclusion

Although rare, aneurysmal complications of KD pose a life-threatening risk but can be effectively managed with CABG, even in pediatric patients, before the onset of left ventricular dysfunction. While LIMA remains the preferred graft, SVGs offer a safe conservative alternative treatment in children when coupled with aggressive antiplatelet therapy.

## Data Availability

The datasets presented in this study can be found in online repositories. The names of the repository/repositories and accession number(s) can be found in the article/Supplementary Material.
